# Adaptive filtering of physiological noises in fNIRS data

**DOI:** 10.1186/s12938-018-0613-2

**Published:** 2018-12-04

**Authors:** Hoang-Dung Nguyen, So-Hyeon Yoo, M. Raheel Bhutta, Keum-Shik Hong

**Affiliations:** 1 School of Mechanical Engineering, Pusan National University, Busan, 46241 Republic of Korea; 2 Department of Automation Technology, Can Tho University, Can Tho, 900000 Vietnam; 3 Department of Computer Science and Engineering, Sejong University, Seoul, 05006 Republic of Korea; 4 Department of Cogno-Mechatronics Engineering, Pusan National University, Busan, 46241 Republic of Korea

**Keywords:** Functional near-infrared spectroscopy (fNIRS), Hemodynamic response (HR), Recursive least squares estimation (RLSE), Exponential forgetting, Real time estimation, State space model

## Abstract

The study presents a recursive least-squares estimation method with an exponential forgetting factor for noise removal in functional near-infrared spectroscopy data and extraction of hemodynamic responses (HRs) from the measured data. The HR is modeled as a linear regression form in which the expected HR, the first and second derivatives of the expected HR, a short-separation measurement data, three physiological noises, and the baseline drift are included as components in the regression vector. The proposed method is applied to left-motor-cortex experiments on the right thumb and little finger movements in five healthy male participants. The algorithm is evaluated with respect to its performance improvement in terms of contrast-to-noise ratio in comparison with Kalman filter, low-pass filtering, and independent component method. The experimental results show that the proposed model achieves reductions of 77% and 99% in terms of the number of channels exhibiting higher contrast-to-noise ratios in oxy-hemoglobin and deoxy-hemoglobin, respectively. The approach is robust in obtaining consistent HR data. The proposed method is applied for both offline and online noise removal.

## Introduction

Functional near-infrared spectroscopy (fNIRS) is a non-invasive optical brain imaging technique that measures oxy-hemoglobin (HbO) and deoxy-hemoglobin (HbR) concentrations in the brain (or body) [1]. A continuous wave fNIRS (CW-fNIRS) system detects a brain activity based on the intensity changes of the detected light while a constant intensity of the incident light is continuously exposed on the brain. The incident light from the emitters placed on the subject’s scalp penetrates several layers of brain tissue (i.e., the scalp, skull, cerebrospinal fluid, gray matter, and white matter), during which some photons are deflected/scattered; pass through the layers, and are detected by a detector (i.e., optode, photodiode) positioned on the scalp approximately at a distance in the range of 0.5–5.5 cm from the emitters [2, 3]. The intensity changes in the detected lights are then utilized to compute the changes of HbO and HbR by using the modified Beer–Lambert law (MBLL) [1, 4–9]. The advantages of the fNIRS technique are non-invasive, inexpensive, quiet, harmless, and portable. It is especially promising for real-time and mobile applications (e.g., daily living environment, intensive aerobic exercises) [10–13]. Recently fNIRS has been shown as appropriate for neuronal activity detection [14–16] and brain–computer interface (BCI) [7, 17–20].

Event-related fNIRS signals are normally contaminated by physiological noises (i.e., heartbeat, respiration, and Mayer waves), extra-cortical physiological noises from the superficial layers, and motion artifacts. This leads to inaccuracies in the obtained cortical activity data. Therefore, it is necessary to remove these noises prior to analyzing the targeted brain functions [21]. Several approaches to reduce these noises were applied in extant studies.

In relation to the motion artifacts that normally originate from the subject’s body movement during the experimentation, various methods including Wiener filtering [22], correlation-based signal correction [23], wavelet transform [24], combined moving average and wavelet [25], an autoregressive model [26], spline interpolation [27], independent component analysis (ICA) [28], targeted principle component analysis (tPCA) [29], and kurtosis-based wavelet filtering [30] were utilized for removal, reduction, and/or correction. Specifically, the wavelet transform [24], tPCA [29], and kurtosis-based wavelet transform [30] are associated with increasing effectiveness when compared with other methods in the reduction of motion artifacts. Least mean squares (LMS) approaches were intended to reduce physiological noises from the superficial scalp and skull layers [31, 32]. Also a recursive least-square estimator (RLSE) was demonstrated in Zhang et al. [33] with fNIRS data generated by Monte Carlo simulation (no experimental data), in which the optode configuration including short- and long- separation channels was utilized to get both superficial and brain tissue components from the five-layer slab model. The RLSE algorithm is faster computationally than the LMS method. In addition, it was demonstrated that a short-separation channel of less than 9 mm was robust to the signal variation in the superficial layer. For investigating noise reduction, various approaches (i.e., band-pass filtering, correlation-based signal improvement, median filtering, Savitzky–Golay filtering, wavelet denoising, and ICA) have been pursued [34].

Recently, physiological noises (approximately 0.1 Hz for Mayer wave, 0.25 Hz for respiration, and 1 Hz for a heartbeat) have been modeled as a sum of sinusoidal functions [10, 35–37] and estimated by using a general linear model (GLM) [10, 38] or an autoregressive moving average model with external inputs [37]. Specifically, Prince et al. [35] proposed a physiological noise model consisting of three sine and cosine functions in which the frequencies of heartbeat, respiration, and Mayer wave were assumed as known. The amplitudes of the sine and cosine functions are estimated by using the extended Kalman filter. In a manner similar to Prince et al. [35], Abdelnour and Huppert [10] used three sinusoidal functions in their physiological noise model. It is noted that Scarpa et al. [36] used the same model of Prince et al. for physiological noise removal although the number of sine/cosine components varied based on the provided data, and it is claimed that the amplitudes of these components (in addition to the Kalman filtering technique) were estimated by using the least-square estimation method [36]. To obtain the corrected signals, the estimated physiological noise signal was subtracted from the acquired signals. Subsequently, the corrected signal of each trial was filtered by a Bayesian approach to improve the accuracy of the HR estimation. Specifically, diverse adaptive filtering approaches [10, 21, 31, 32, 35, 36, 38] are used for other noise reductions.

An emitter-detector pair of distance less than 1 cm is termed as a short-separation (SS) channel. This type of SS channel is used to acquire the noise in the superficial layer because the detected light in this case passes only through the superficial layer and does not reflect any cognitive activity [36, 39–44]. Saager and Berger [39] and Saager et al. [39, 41] suggested a method to reduce superficial noises by subtracting one SS measurement from a long-separation measurement. Additionally, Zhang et al. [33] demonstrated that a RLSE based adaptive method significantly reduced the superficial noises. In their study, the superficial noise estimated through the coefficient of one SS channel within the framework of RLSE. Recently, the long-separation measurement has been modeled as a linear form consisting of the expected HR and SS measurement [40, 42, 43]. It is noted that the weights of canonical functions (i.e., a combination of 15 Gaussian functions) and the amplitudes of SS data were estimated by using the Kalman filter approach. Their proposed method revealed a significant improvement in both HbOs and HbRs when compared to those obtained by the traditional adaptive filter or the standard GLM model. Additionally, Sato et al. [44] estimated the extra-cortical signal by using the GLM model, and subsequently, the corrected HR was obtained by subtracting the estimated extra-cortical signal from the measured long-separation channel signal.

Clearly, fNIRS data are contaminated by extra-cortical noises from the extra-cortical layers that occur when the light travels through the extra-cortical layers (i.e., scalp, skull, cerebrospinal fluid) prior to/after reaching the cortical layers (i.e., gray and white matter). Superficial noises are exposed in either single-SS [42] or double-SS measurement [43]. The fNIRS data obtained by the SS detectors (emitter-detector pair distance: 0.5 cm) contains extra-cortical physiological noises while data obtained by the long-separation detectors (emitter-detector pair distance: approximately 3 cm) contains HR information for both extra-cortical and cortical tissues [39, 42]. The SS measurements have been included in the GLM model involving the expected brain HR [42, 43], and the proposed Kalman estimator method obtained the efficiency of noise reduction up to 50 and 100% [42]. Additionally, Gagnon et al. [43] demonstrated that the use of the double-SS measurement reduces noises in the HbO and HbR by 59% and 47%, respectively.

In the fNIRS field, the canonical HR functions were usually generated by a combination of gamma functions [45, 46]. However, the state-space model developed in [46] is specifically convenient when compared with the use of canonical HR functions in which the impulse HR for an impulse stimulation at a specific cortex was reconstructed as a state-space equation by using the subspace identification method. It should be noted that the shapes of impulse HRs in individual cortices are different. Thus, the expected HR for an arbitrary stimulus is generated online (or in real-time).

In the study, we propose an adaptive-filter-based method to reduce physiological and superficial noises in fNIRS data. The mathematical model for filtering is a linear form comprised of the following four main components: the expected HR, SS data, the sum of sinusoidal functions representing physiological noises, and the baseline drift. The expected HR is generated with given stimuli by using the state-space model developed in [46]. The SS data (emitter-detector distance: 0.5 cm) are utilized to obtain the extra-cortical noise from the superficial layer. The physiological noises are modeled as a sum of three sinusoidal functions by following the method developed in [10, 35, 36]. In order to estimate the baseline value, the corresponding element in the regression vector is set to unity although its coefficient *b*_0_ (i.e., *b*_0_ × 1) is estimated (see “Methodology” section for more details). The unknown parameter vector in the proposed model is estimated by using the RLSE with an exponentially forgetting factor. Finally, the efficacy of the proposed method is demonstrated by using experimental right-finger-movement fNIRS data obtained from the left motor cortex. Our experimental results indicate that the proposed method significantly reduces physiological and superficial noises when compared with the existent approaches. Thus, it is possible to apply the proposed method to remove noises in both offline and online cases.

## Methodology

### Theory (brain activity model)

**Fig. 1:**
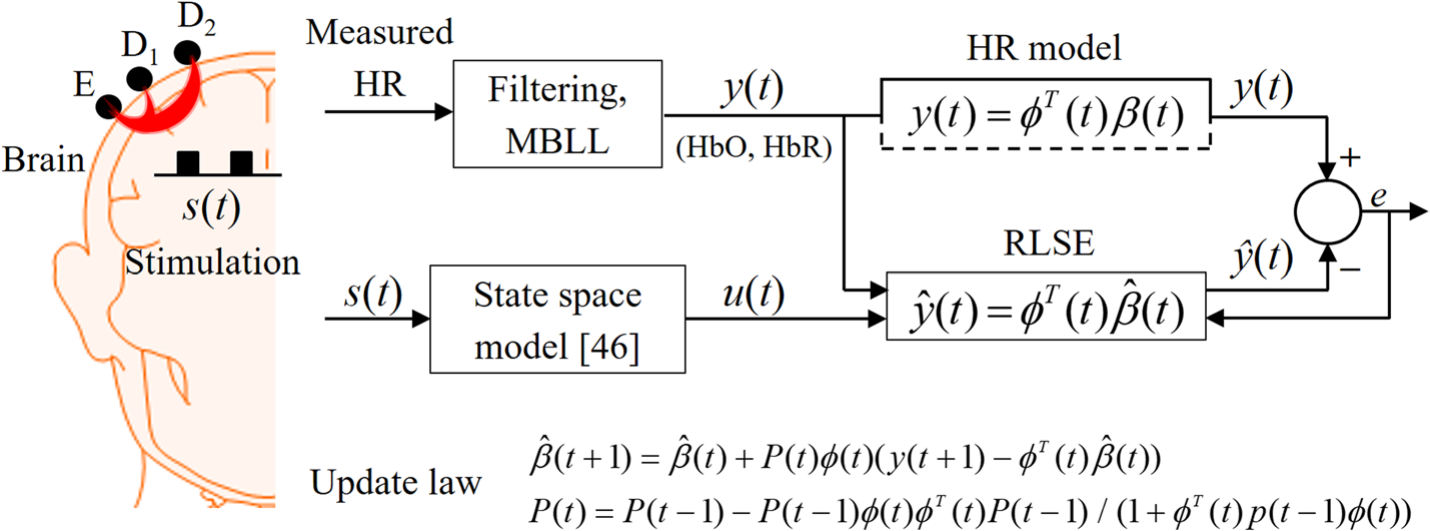
Schematic of workflow for processing hemodynamic responses (HRs): E-D_1_/E-D_2_ illustrate short-/long-separation channels, respectively (*MBLL* modified Beer–Lambert law)

In this study, the hemodynamic response caused by a brain activity is modeled in a linear form as follows:

1 $$y(t) = a_{1} u(t) + a_{2} \Delta u(t) + a_{3} \Delta^{2} u(t) + a_{4} y_{\text{SS}} (t) + \;\sum\limits_{m = 1}^{q} {b_{m} \sin (2\pi f_{m} t) + b_{0} y_{\text{b}} + \varepsilon (t),}$$

where *t* denotes the discrete time, *y*(*t*) represents the measured HR signal acquired by a pair of long-separation optodes (for e.g., E-D_2_ pair in Fig. 1) at time *t*; *u*(*t*) denotes the expected HR generated by a state-space model [46]; Δ*u*(*t*) and Δ^2^*u*(*t*) denote the first and second derivatives of *u*(*t*), respectively; $y_{\text{SS}} (t)$ (in which the subscript SS refers to short-separation) denotes the extra-cerebral physiological noise in the superficial layer (e.g., the channel E-D_1_ in the emitter side in Fig. 1); *f*_*m*_ denotes the frequencies of physiological noises; *q* denotes the total number of physiological noises (in this study, *q* is set to 3); *y*_b_ denotes the term introduced to correct the baseline per trial; *ε*(*t*) denotes the white Gaussian noise, and *a*_1_, *a*_2_,… *a*_4_, *b*_1_, *b*_2_,…, *b*_*q*_, and *b*_0_ denote unknown coefficients that are to be estimated. In extant studies, three important frequencies of physiological noises are 1 Hz (cardiac), 0.25 Hz (respiratory), and 0.1 Hz (low frequency arterial blood pressure oscillation) [47, 48]. In the study, these three frequencies were estimated with the *fft* function available in MATLAB (MathWorks Inc.) from the data of the initial resting state data.

Equation (1) is rewritten as follows:

2 $$y(t) = \varphi^{T} (t)\beta (t) + \varepsilon (t),$$

where *ϕ*(*t*) = [*u*(*t*) Δ*u*(*t*) Δ^2^*u*(*t*) *y*_SS_(*t*) sin(2π*f*_1_*t*) sin(2π*f*_2_*t*) … sin(2π*f*_*q*_*t*) 1]^*T*^ denotes the regression vector, *β*(*t*) = [*a*_1_ *a*_2_ … *a*_4_ *b*_1_… *b*_*q*_ *b*_0_]^*T*^ denotes the unknown coefficient vector, and the superscript *T* denotes the transpose operator. Additionally, *β*(*t*) is estimated by the RLSE approach. According to previous works in the control field, the RLSE algorithm gives a good performance in parameter estimation [49–52] and could be utilized in real-time applications [53–56]. Therefore, this algorithm is chosen to estimate unknown parameter vector *β*(*t*). Thus, by using Eq. (2), the estimated brain activity is represented as follows:

3 $$\hat{y}(t) = \varphi^{T} (t)\hat{\beta }(t) + \varepsilon (t),$$

where ^ denotes the estimated values. We denote $$\hat{\beta }(t) = \left[ {\begin{array}{*{20}c} {\hat{a}_{1} } & {\hat{a}_{2} } & \ldots & {\hat{a}_{4} } & {\hat{b}_{1} } & \ldots & {\hat{b}_{q} } & {\hat{b}_{0} } \\ \end{array} } \right]^{T}$$ as the estimated parameter vector, which is obtained by optimizing the cost function $J(\beta ,t) = \frac{1}{2}\sum\nolimits_{k = 1}^{t} {\lambda^{t - k} (y(k) - \varphi^{T} (k)\beta )^{2} }$ (where λ denotes the forgetting factor). Its recursive update law is given as follows [57]:

4 $$\begin{aligned} \hat{\beta }(t + 1) & = \hat{\beta }(t) + P(t)\varphi (t)(y(t + 1) - \varphi^{T} (t)\hat{\beta }(t)), \\ P(t) & = P(t - 1) - P(t - 1)\varphi (t)\varphi^{T} (t)P(t - 1)\;/\;(1 + \varphi^{T} (t)P(t - 1)\varphi (t)) \\ \end{aligned}$$

where *P*(*t*) denotes the covariance matrix. We assume that $e(t) = y(t) - \varphi^{T} \hat{\beta }(t)$ is the estimation error.

Figure 1 illustrates a schematic for brain activity estimation. The detectors’ light intensities are acquired by dual-wavelength CW-fNIRS (i.e., 760 and 830 nm). We assume that *s*(*t*) is the (arbitrary) stimuli that activates a certain brain region, which corresponds the input signal to the state-space model. The output of the box is termed as the expected/desired HR because we expect the HR to exhibit this type of a response. Additionally, its first and second derivatives are used in Eq. (1) as components in the regression vector. The difference between the measured data and the estimated data (i.e., *e*(*t*)) was utilized in updating the parameter vector $\hat{\beta }(t)$, see (4). In the present study, the proposed method was applied to detect right-finger-movements in the left motor cortex.

Thus, to illustrate the necessity to estimate the frequencies of physiological noises, two cases are discussed (i.e., *f*_*m*_ vs. $\hat{f}_{m}$, fixed and estimated). First, to eliminate a possible contribution of superficial noises in the comparison, the SS channels are ignored. Therefore, the two following models are utilized.

5 $$\hat{y}(t) = \hat{a}_{1} u(t) + \hat{a}_{2} \Delta u(t) + \hat{a}_{3} \Delta^{2} u(t) + \sum\limits_{m = 1}^{q} {\hat{b}_{m} \sin (2\pi f_{m} t) + \hat{b}_{0} y_{\text{b}} + \varepsilon (t)} ,$$

6 $$\hat{y}(t) = \hat{a}_{1} u(t) + \hat{a}_{2} \Delta u(t) + \hat{a}_{3} \Delta^{2} u(t) + \sum\limits_{m = 1}^{q} {\hat{b}_{m} \sin (2\pi \hat{f}_{m} t) + \hat{b}_{0} y_{\text{b}} + \varepsilon (t)} .$$

It is noted that the fixed frequencies of *f*_*m*_ (0.1 Hz, 0.25 Hz, and 1 Hz) in (5) were obtained from [10, 37] and the estimated frequencies $\hat{f}_{m}$ in (6) were obtained from the measured data during the resting state by using the *fft* function available in MATLAB.

The main objective of the current study is to reduce both physiological and superficial noises. In addition, $\hat{a}_{1} u(t) + \hat{a}_{2} \Delta u(t) + \hat{a}_{3} \Delta^{2} u(t)$ from (3) is extracted for the estimated HR. It is noted that the physiological noise frequencies are estimated on-line and are subsequently included in (3). The parameters are estimated using the RLSE approach. Both numerical and real experimental data are processed. The RLSE method is utilized to estimate the weights of the linear combination of the expected HR and physiological noises (i.e., heart and respiratory waves).

### Numerical validation of RLSE

**Fig. 2:**
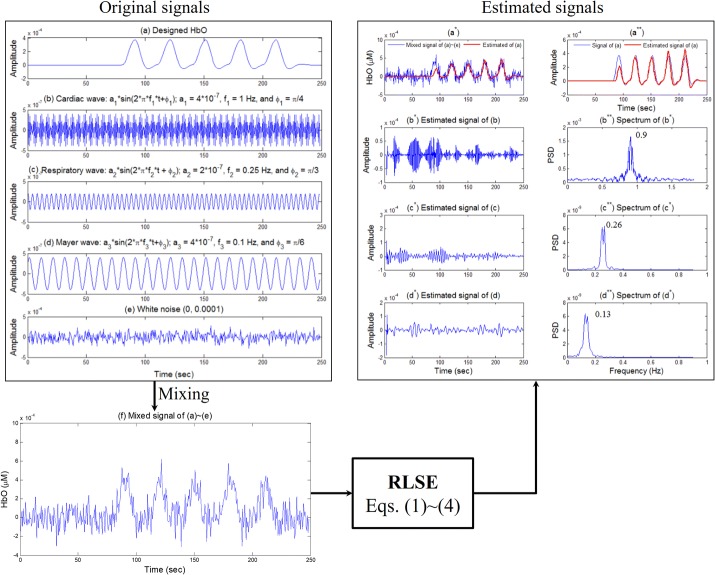
Numerical verification of the proposed recursive least squares estimation (RLSE) method with an exponential forgetting factor to extract physiological noises: **a** Expected HbO, **b** cardiac, **c** respiratory, **d** Mayer wave, and **e** white noise with zero mean and standard deviation of 10^−4^, **f** mixed signal of **a**–**e**; a^*^ a comparison of **f** and the estimated signal of **a**; b^*^–d^*^ the estimated signals of **b**–**d**; b^**^–d^**^ the spectra of b^*^–d^*^, respectively

Numerical simulations are performed to validate the appropriateness of using of the RLSE algorithm for decoding brain hemodynamics. First, five related signals are intentionally mixed, see Fig. 2: a the expected HbO signal, b a cardiac signal, c a respiratory signal, d a typical Mayer wave, and e a white noise with zero mean and standard deviation of 10^−4^. The mixed signal of five signals is shown in Fig. 2f. In the process, the values of the amplitudes and frequencies of physiological noises were adopted from [36]. The proposed method of Eqs. (1)–(4) is applied to the mixed signal to estimate the original five signals as shown in Fig. 2a^*^ and a^**^. Specifically, the estimated HbO (the red thick curve) in Fig. 2a^**^ was obtained by computing the first three terms, i.e., $\hat{a}_{1} u(t) + \hat{a}_{2} \Delta u(t) + \hat{a}_{3} \Delta^{2} u(t)$. Figures 2b^*^–d^*^ correspond to the estimated physiological noises of Fig. 2b–d, respectively. Figures 2b^**^–d^**^ denote the spectra of Fig. 2b^*^–d^*^, respectively. As shown in Fig. 2a^**^, the estimated HbO (the red curve) is gradually updated to the desired HbO (the blue curve in Fig. 2a). Furthermore, the estimated frequencies of cardiac (0.9 Hz), respiratory (0.26 Hz), and Mayer (0.13 Hz) waves (see Fig. 2b^**^–d^**^) are sufficiently close to the known ones in Fig. 2b–d, respectively. The result demonstrates that the proposed method effectively extracts the correct HbO and the physiological noises.

### Experimental paradigm design and verification

**Fig. 3:**
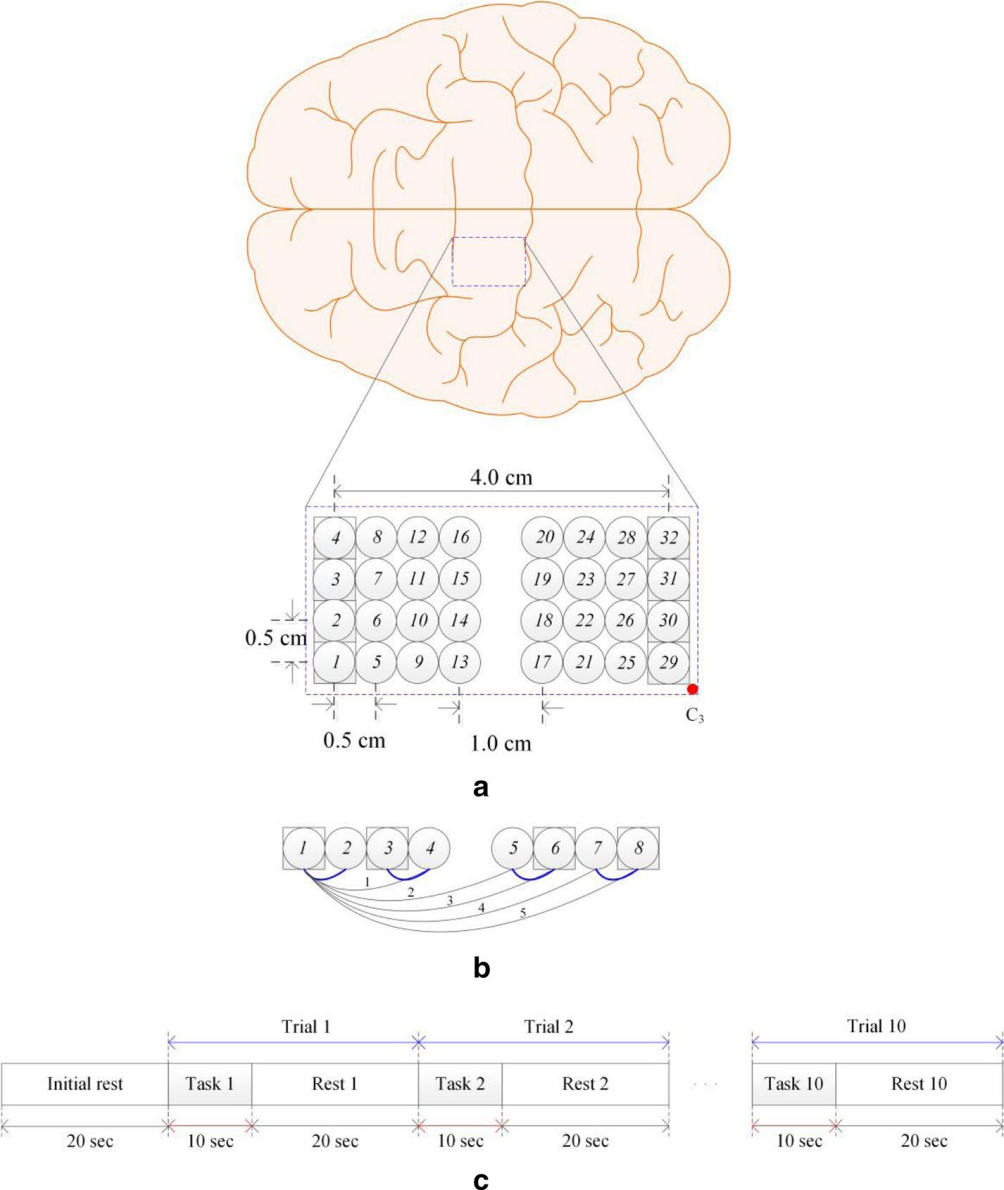
**a** Bundled-optode configuration of 40 channels to detect right-finger movements in the left motor cortex (squares and circles represent emitters and detectors, respectively); **b** an example of short-/long-channel measurements (short: *1*–*2*, *3*–*4*, *5*–*6*, *7*–*8*; long: *1*–*4*, *1*–*5*, *1*–*6*, *1*–*8*, etc.; for instance, if Ch. 1 is composed of emitter *1* and detector *4*, pairs *1*–*2* and *3*–*4* are used as a SS channel for Ch.1′s noise removal); and **c** experimental paradigm (five trials for thumb and little-finger movements each, which were randomly performed)

Figure 3a shows an optode arrangement for verifying the developed method in which multiple emitters and detectors are placed side by side (as a bundle) to form multiple short- and long-separation channels [58, 59] Additionally, according to Zhang et al. [33], since short-separation less than 9 mm was robust in various layers, all short-separation channels with 5 mm distance are therefore configured in the current study. And each optode is used as either an emitter or a detector. Figure 3b depicts a set of short- and long-separation channels: The squares in Fig. 3b represent emitters, and the circles are detectors. Specifically, the pairs *1*–*2*, *3*–*4*, *5*–*6*, and *7*–*8* are SS channels (0.5 cm apart). The long separation channels (i.e., approximately 1.5–4.0 cm apart) are numbered in plain text underneath, i.e., *1*–*4*, *1*–*5*, *1*–*6*, *1*–*7*, and *1*–*8*.

In Ch. 1, which is composed of emitter *1* and detector *4*, two SS measurements are considered (i.e., *1*–*2* or *3*–*4*). Either measurement is included in Eq. (1). Five long-separation channels are considered when optode *1* emits light. Similarly, an additional five channels are formed from the right to the left when optode *8* shoots light. Therefore, ten channels are created in each row. In the study, only a total of 40 channels (eight channels for five distances: 1.5, 2.5, 3.0, 3.5, and 4.0 cm) were included in computation owing to the computation time constraints. Figure 3c illustrates the experimental paradigm to detect right-finger-movement brain activity.

#### Study participants

Five healthy male participants (mean age 36.2; range: approximately 33–37 years) with shaved hair were invited to perform experiments (thumb and little-finger movements) involving the left motor cortex. None of the subjects exhibited any neurological impairments or mental disorders. Four of the subjects were right-handed. To eliminate any interference from the external noise, the experiments were conducted in a dark and quiet room. The subjects were asked to sit comfortably on a chair and not to move their body during the experiment. Prior to starting the experiment, the subjects were carefully trained in how to move their fingers.

Figure 3c illustrates the experimental paradigm: An experiment comprised of ten trials of little-finger and thumb movements; and a trial consisted of a 10 s task and a 20 s rest. After a 20 s initial rest period prior to the first trial, each subject was asked to move their fingers (i.e., flexion and extension) for 10 s by watching the screen in which each finger randomly appeared five times. Therefore, an experiment corresponded to a total of 320 s. To increase the brain activity during the 10 s task period, the subjects were asked to move their (right) little-finger/thumb as fast as possible without paying attention to the number of flexions/extensions and to relax during the 20 s rest period. A laptop computer with a 15-inch screen was utilized to display pictures indicating each finger. The distance between the subject’s eyes and the laptop screen was adjusted as approximately 60 cm such that the subjects could clearly see the indicated fingers. The subjects were also instructed to keep their eyes open during the experiments. During the rest period, a black screen was displayed to relax the subjects’ eyes.

The optodes in Fig. 3a were positioned over the subject’s left motor cortex to record the HRs to the right finger movements. Prior to the experiments, the nature of the experimental procedures was clearly explained to the subjects. All the experiments in the study were performed following the guidelines of the Institutional Review Board of Pusan National University, and informed consent were obtained from all the subjects based on the Declaration of Helsinki.

#### Equipment and data conversion

Dual-wavelength continuous-wave fNIRS (DYNOT, NIRx, USA) was utilized to measure the brain’s hemodynamic responses. The intensities of the detected light were converted to hemoglobin concentration changes by using the MBLL. A total of 40 channels over the left motor cortex were configured at a sampling rate of 1.81 Hz. In the present study, the recorded fNIRS data drifted in time [45, 58, 59], and thus a baseline-correction method was applied. Specifically, a 4th order polynomial was fit to the data, and the obtained curve was subtracted from the original data to remove the drift [59, 60].

### Contrast-to-noise ratio

The contrast-to-noise ratio (CNR) denotes the weighted difference between the mean of the signal during the task and that during the rest period [23, 59]. To validate our proposed method, the CNRs were used to check the signal-to-noise ratio, since a high CNR value indicates a high ratio of the signal upon the task relative to that to noise. The CNR is computed as follows:

7 $${\text{CNR}} = \frac{{{\text{mean}}(task) - {\text{mean}}(rest)}}{{\sqrt {\text{var} (task) + \text{var} (rest)} }},$$

where “task” indicates the task period and “rest” denotes the rest period prior to finger movement. In the present study, the “task” window was set to a duration of approximately 4–14 s, and the “rest” window was set to a duration of approximately to -6–0 s from the onset.

## Results and discussion

### Physiological noises during the rest state

**Fig. 4:**
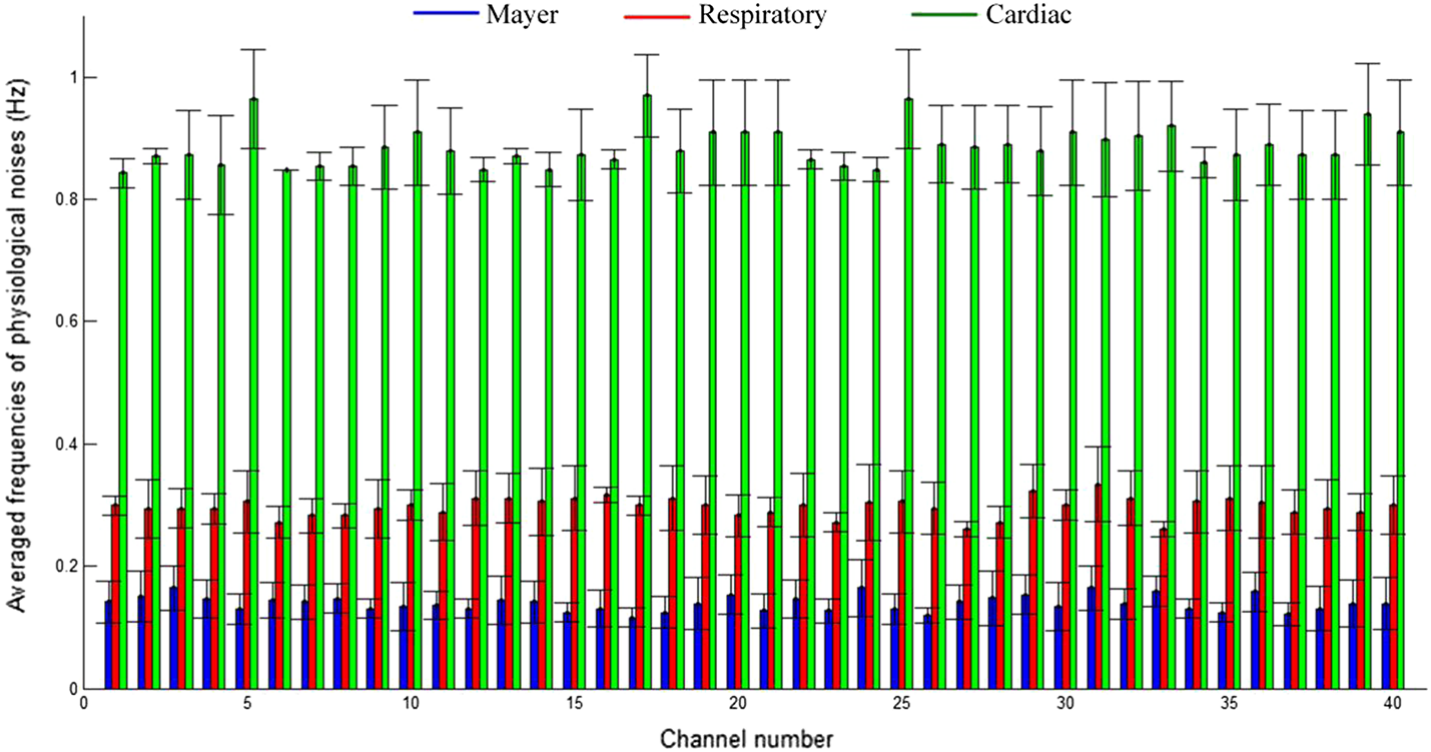
Averaged frequencies (over five subjects) of all 40 channels during the initial 20 s resting period prior to the first trial

Figure 4 compares the average frequencies of Mayer, respiratory, and cardiac signals during the initial 20 s resting period (see Fig. 3c) over 5 subjects. As observed, the averaged frequencies (Mayer, respiratory, cardiac: 0.14, 0.3, and 0.89 Hz, respectively) are extremely consistent throughout the channels. The result also agrees with those in previous studies [47, 48, 61]. However, variations exist per subject (and per time, for e.g., morning and afternoon), and thus, these frequencies are estimated online for each experiment by using the initial resting state data. Finally, the estimated frequencies were reflected as shown in (3).

### Verification of the proposed method

Our main objective involved reducing the physiological and superficial noises and subsequently extracting the correct HR from fNIRS data. In extant studies, Abdelnour and Huppert [10] proposed a brain activity model including two main components, namely the expected HR and the sum of three sinusoidal functions representing the physiological noises. Their proposed method demonstrated a significant reduction in the physiological noises although the frequencies of those noises were assumed constant. In the study, we use a linear model in which the physiological noise frequencies are estimated online during the initial resting state (prior to the first trial). Thus, by using the *fft* function, they are computed at the initial 20 s resting period per channel. Additionally, those estimated frequencies are included into the *f*_m_ in Eq. (3).

A SS measurement records the extra-cortical noise in the superficial layer while a long-separation channel includes the brain HR from both the cerebral cortex and the superficial layer [41–43, 62]. In the current study, SS measurements were utilized as reference channels to remove noises in the superficial layer.

**Fig. 5:**
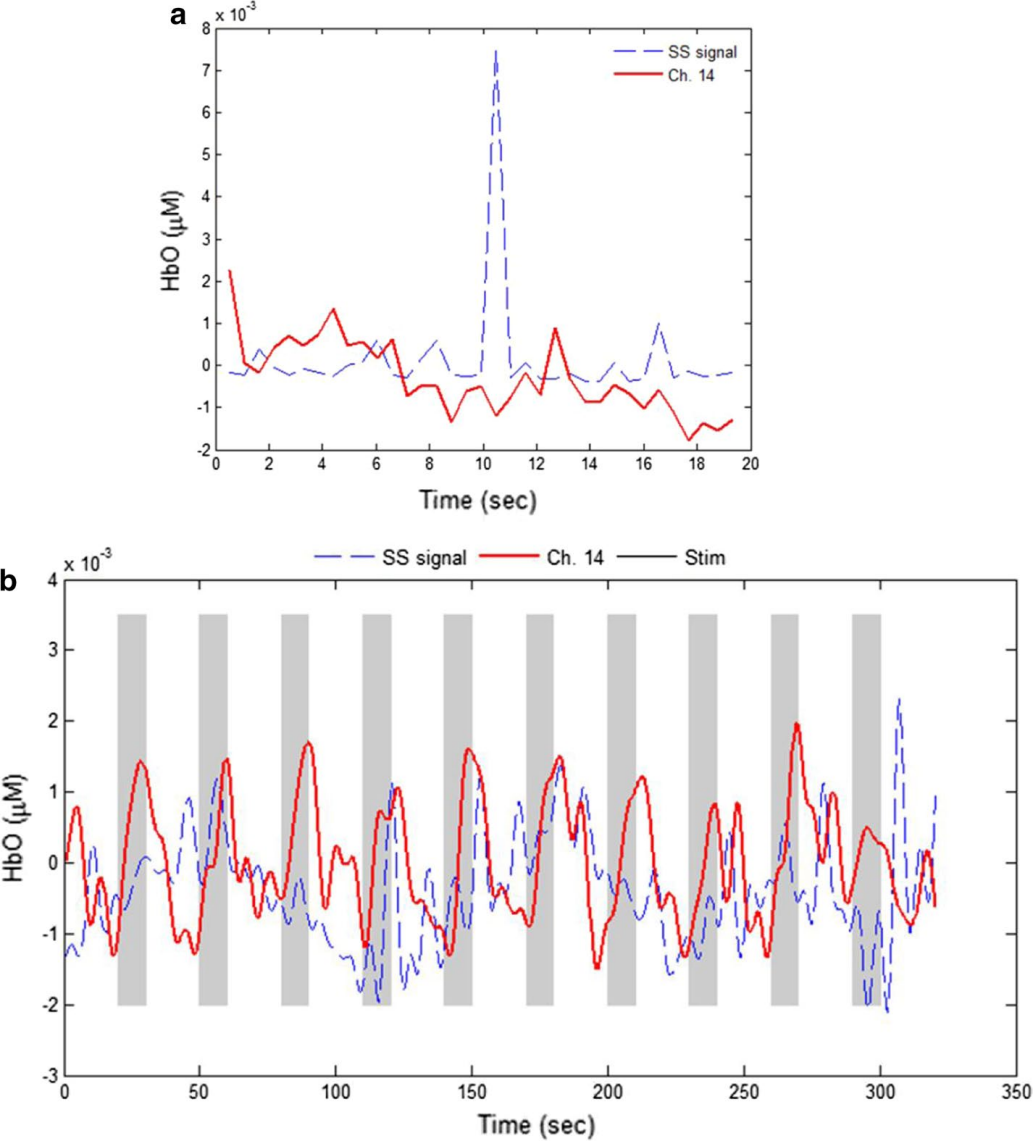
Comparison of a SS channel and an active long-separation channel (Sub. 2, Ch. 14 was formed by optodes 4 and 28 in Fig. 3a): **a** Raw data during the initial 20 s resting period (the peak in blue curve is due to head movement). Correlation (r = − 0.15) of raw short- and active long-separation channel in the 20 s resting state. **b** Band-pass-filtered signals (cut-off frequencies: 0.01 and 0.15 Hz) of both data for the entire experimental period. Correlation (r = 0.099) of short- and active long-separation channel

To investigate whether the extra-cortical noise and the physiological noise are related, the correlation coefficients between the raw SS signals and the active long-separation channel data during the initial 20 s resting period were computed. In most channels, the correlation coefficients were less than 0.34. Figure 5a depicts an example of SS data and a long-separation channel (Ch. 14, Sub. 2): In this case, the correlation coefficient was − 0.15. The peak(s) in the dashed line is due to the shift of an optode during the experiment. Ch.14 was specifically chosen because the correlation coefficient between the channel and its short-separation channel was the lowest with respect to all the channels. Additionally, in most cases, the correlation coefficients between the filtered SS data and the active long-separation signals were less than 0.38. Figure 5b compares the entire data (320 s) of a SS signal and Ch. 14 of Sub. 2 in which the correlation is only 0.099. Thus, it is concluded that the extra-cortical noise and the physiological noises are not correlated. The experimental data confirmed that the SS data only contained the extra-cortical noise and did not include brain-activity related physiological noises.

### Physiological noises between known and estimated frequencies

To investigate the effectiveness of physiological noise frequency estimation, we compared the two brain activity models in (5) and (6), respectively. In (5), three frequencies (i.e., 0.1 Hz, 0.25 Hz, and 1 Hz) corresponded to fixed constants in three sinusoidal functions. However, as shown in (6), the frequencies were estimated online from the initial 20 s resting-state period, and the estimated values per experiment were then used in the RLSE method.

**Fig. 6:**
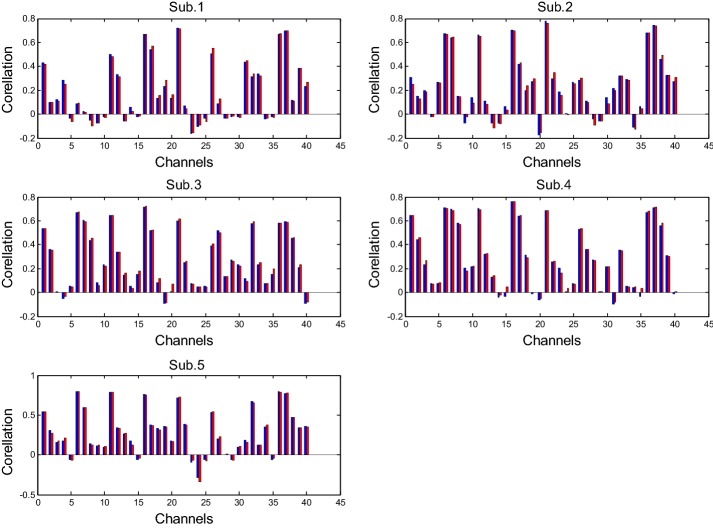
Comparison of correlation coefficients between the desired HRs and the estimated HRs (blue bars: fixed frequencies, red bars: estimated frequencies): For Sub. 1, the number of red bars that exceed the number of blue bars is 22 (out of 40), and this results in 55%

Figure 6 compares the correlation coefficients between the desired HR and the estimated HR that was obtained by using the fixed frequencies (blue bars) and the estimated physiological frequencies (red bars) over 40 channels and 5 subjects. The results show that the percentage improvement in terms of the number of improved channels of Subs. 1–5 by using the estimated frequencies over the fixed ones correspond to 55, 30, 47.5, 45, and 47.5%, respectively. Thus, this indicates that the estimation of the physiological noise frequency did not result in a significant improvement.

**Fig. 7:**
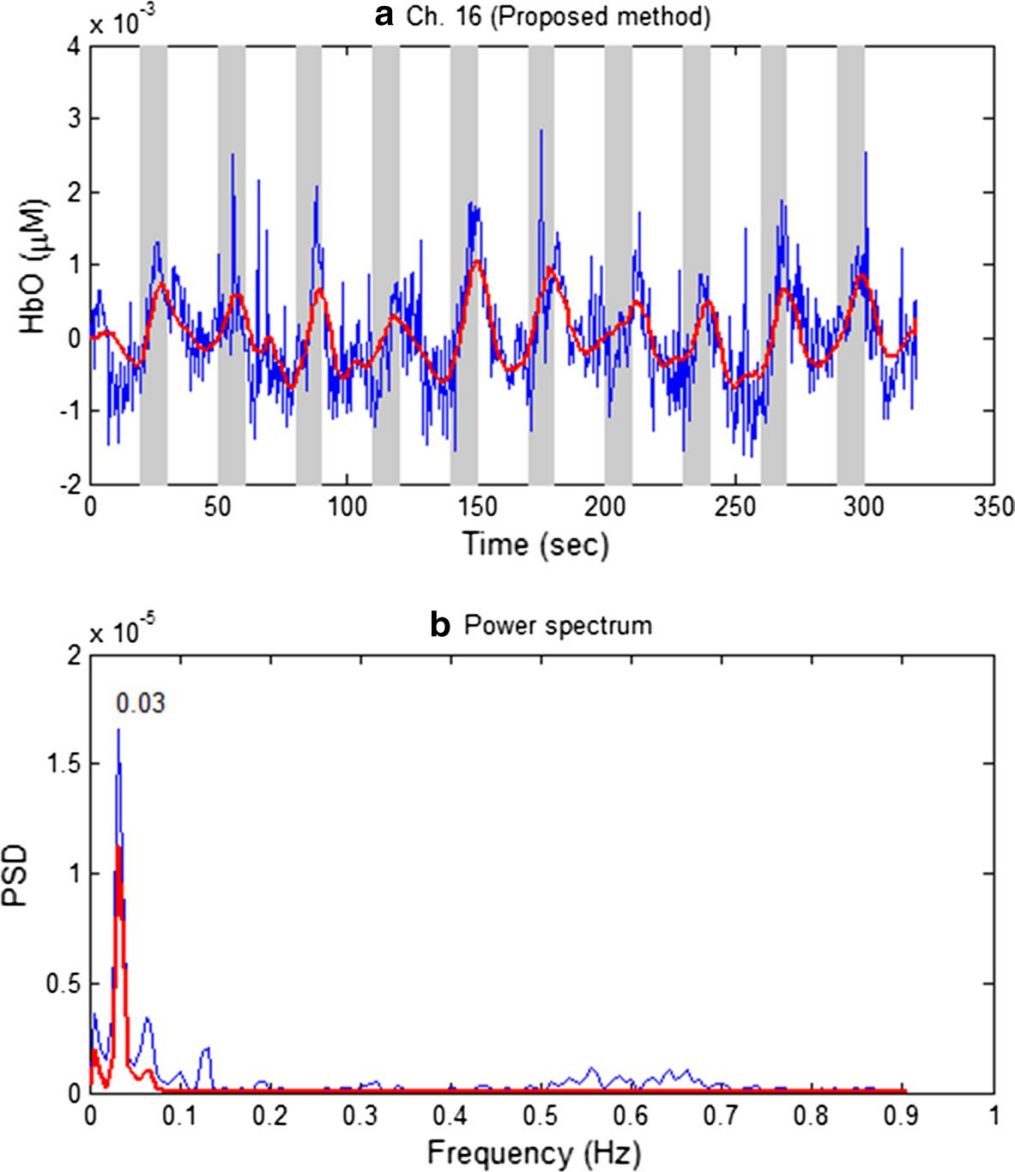
**a** Raw HbO data (blue curve) relative to the estimated HbO (red thick curve) by using the proposed method, **b** power spectra of **a** (Ch. 16, Sub. 2)

Figure 7 plots the estimated HbO of a representative channel (Ch. 16, Sub. 2) by using our proposed method in which Ch. 16 was created by optodes 5 and 17. Figure 7a illustrates the raw HbO data (blue curve) and the estimated data (red thick curve, $\hat{y}(t)$ in Eq. (3)). Figure 7b compares the power spectra of both raw and filtered HbOs approximately within the 0–1 Hz range. As shown in Fig. 7b, the proposed method significantly reduced the physiological noises.

**Fig. 8:**
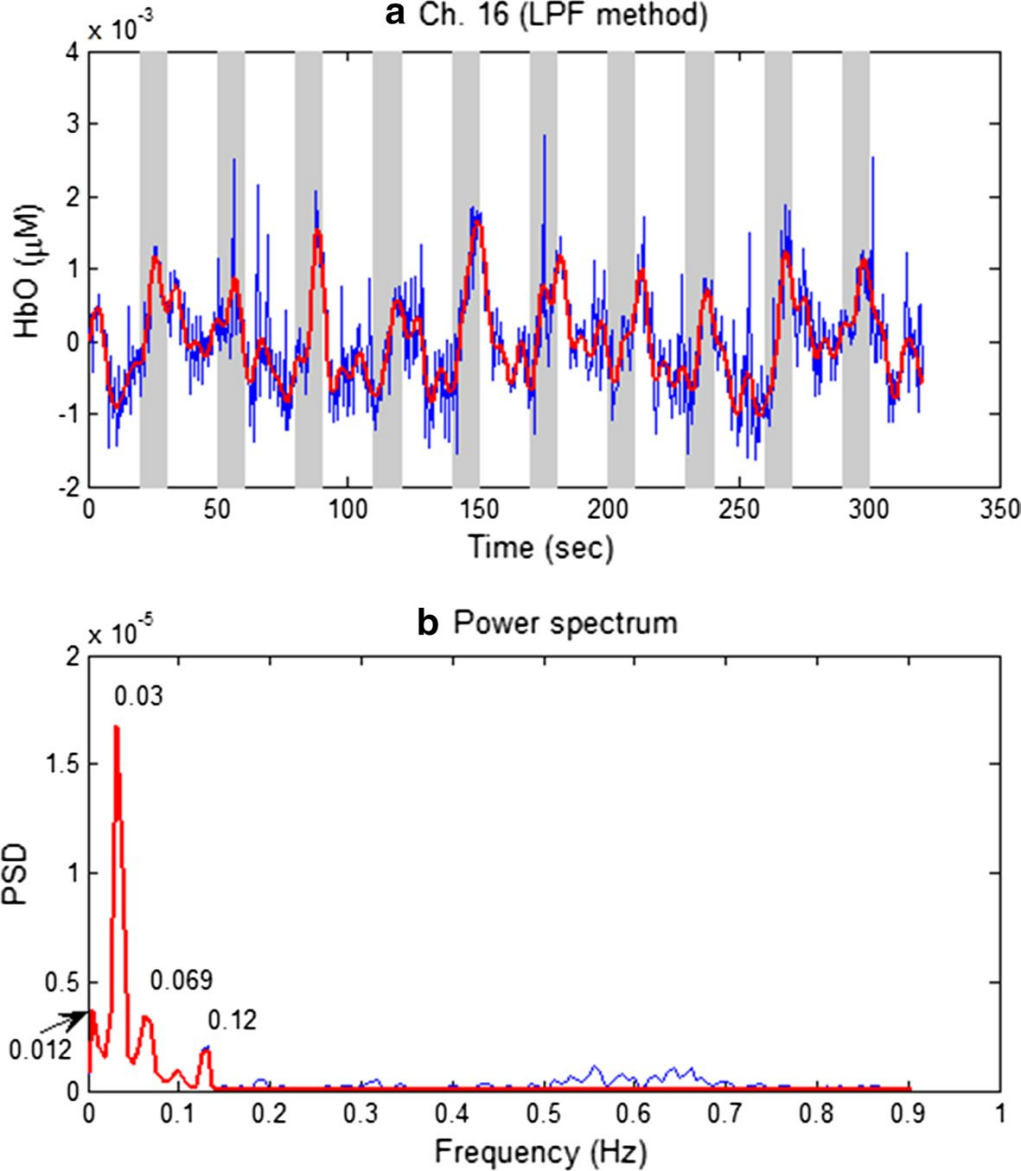
**a** Raw HbO data (blue curve) relative to the low-passed filtered HbO (thick red curve) with 0.15 Hz cut-off frequency, **b** the power spectra of **a** that shows the existence of physiological noises

The proposed method was compared with a conventional method. The low-pass filtering (LPF) approach was popular, and thus a cut-off frequency of 0.15 Hz was applied to the raw data in Fig. 7a: The thick red curve in Fig. 8a denotes the LPF-ed HbO, and its power spectra is shown Fig. 8b. The result reveals that it was not possible to eliminate the physiological noises at 0.012, 0.069, and 0.12 Hz frequencies, by using the LPF method.

**Fig. 9:**
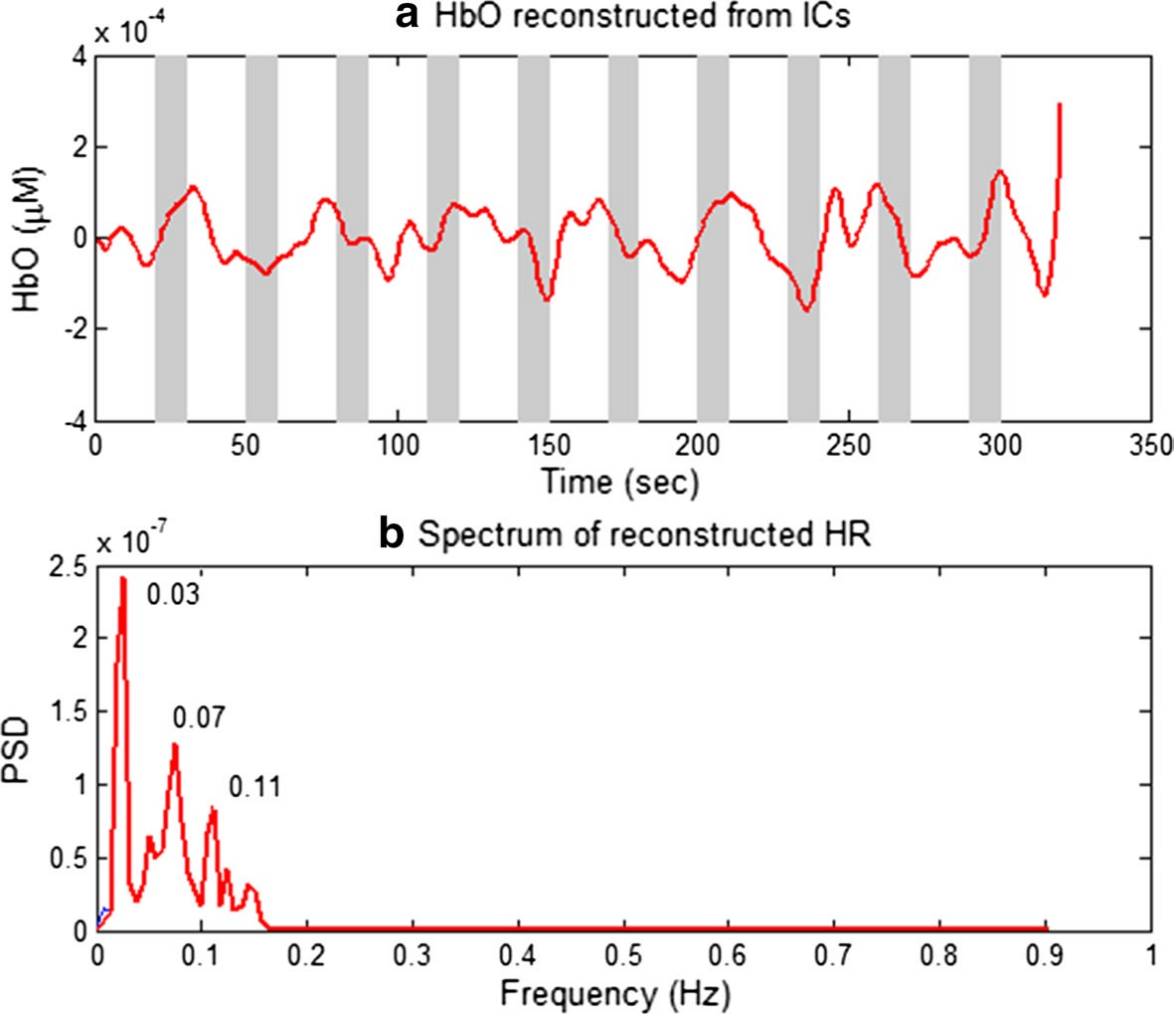
**a** A reconstructed HbO (red thick line) by using the ICA approach (Sub. 2), **b** the spectrum of **a**: ICA decompositions were run 10 times; and the reconstructed one is the one with the highest *t*-value in each trial

For further validation, an ICA based approach was utilized to reconstruct the HR [45]. The task of the ICA approach involved recovering the unknown source signals from the measured fNIRS data. In the process, the number of source signals was assumed as equal to the number of measured signals [63]. The steps of ICA included preliminary whitening of the measured data and estimation of orthogonal ICA transform to obtain the weight vector of all channels. Finally, the independent component (IC) or source signals were estimated from the estimated weight vector and measured data (see [45, 63, 64] for more details). The ICA decomposition was performed several times to yield similar ICs [64]. Figure 9a shows a reconstructed HbO by the ICA approach in which the data of 40 long-separation channels (Sub. 2) are used. As shown in Fig. 9b, the noise frequencies of 0.07 and 0.11 Hz were not eliminated.

**Fig. 10:**
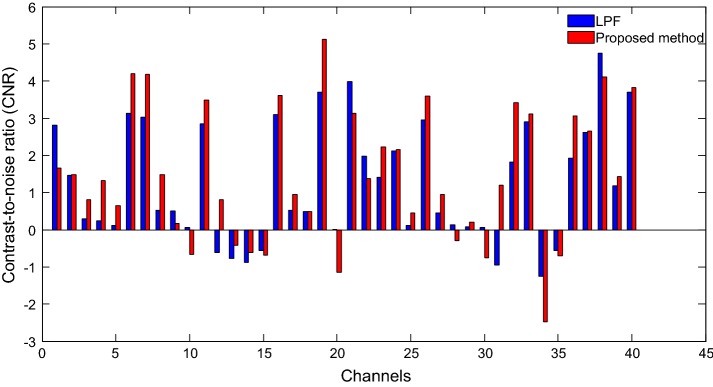
Comparison of CNRs of the HbOs obtained by the LPF approach (blue bars) and the proposed method (red bars) (Sub. 2): The proposed method shows that the number of channels exhibiting improved CNR is higher

**Fig. 11:**
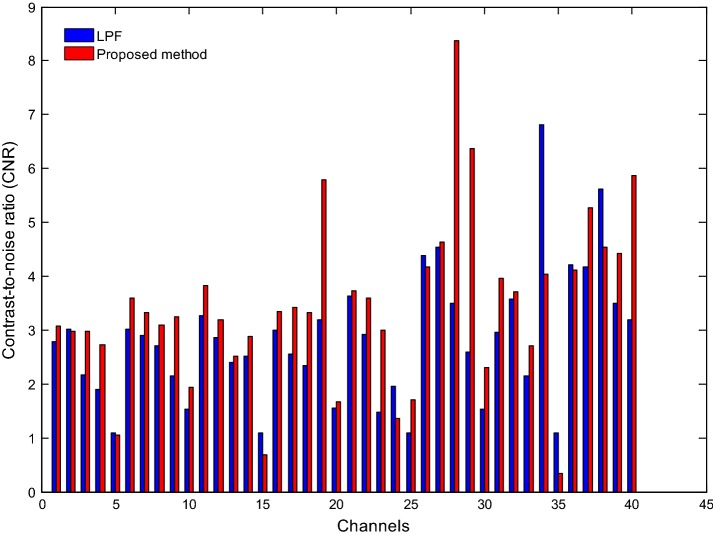
Comparison of CNRs of the HbRs obtained by the LPF approach (blue bars) and the proposed method (red bars) (Sub. 2)

To further validate the proposed method, CNRs in Eq. (7) were compared for both the LPF method and the proposed approach. In the process, the time windows approximately in the range of 4–14 s and − 6–0 s were assigned as “task” and “rest”, respectively. Figure 10 compares the CNRs of the HbOs obtained by two methods: LPF and the proposed method (Sub. 2). As shown, the CNRs of the proposed method (red bars) exceeded those of the LPF method (blue bars). Additionally, the CNRs of the HbRs obtained by the two approaches are shown in Fig. 11. The results indicated that CNRs of the proposed method exceeded those computed by the LPF method in all channels. The result demonstrates that the proposed method extracted the expected HR more precisely by significantly reducing noise.

### Comparison with/without the sum of sinusoidal functions

**Fig. 12:**
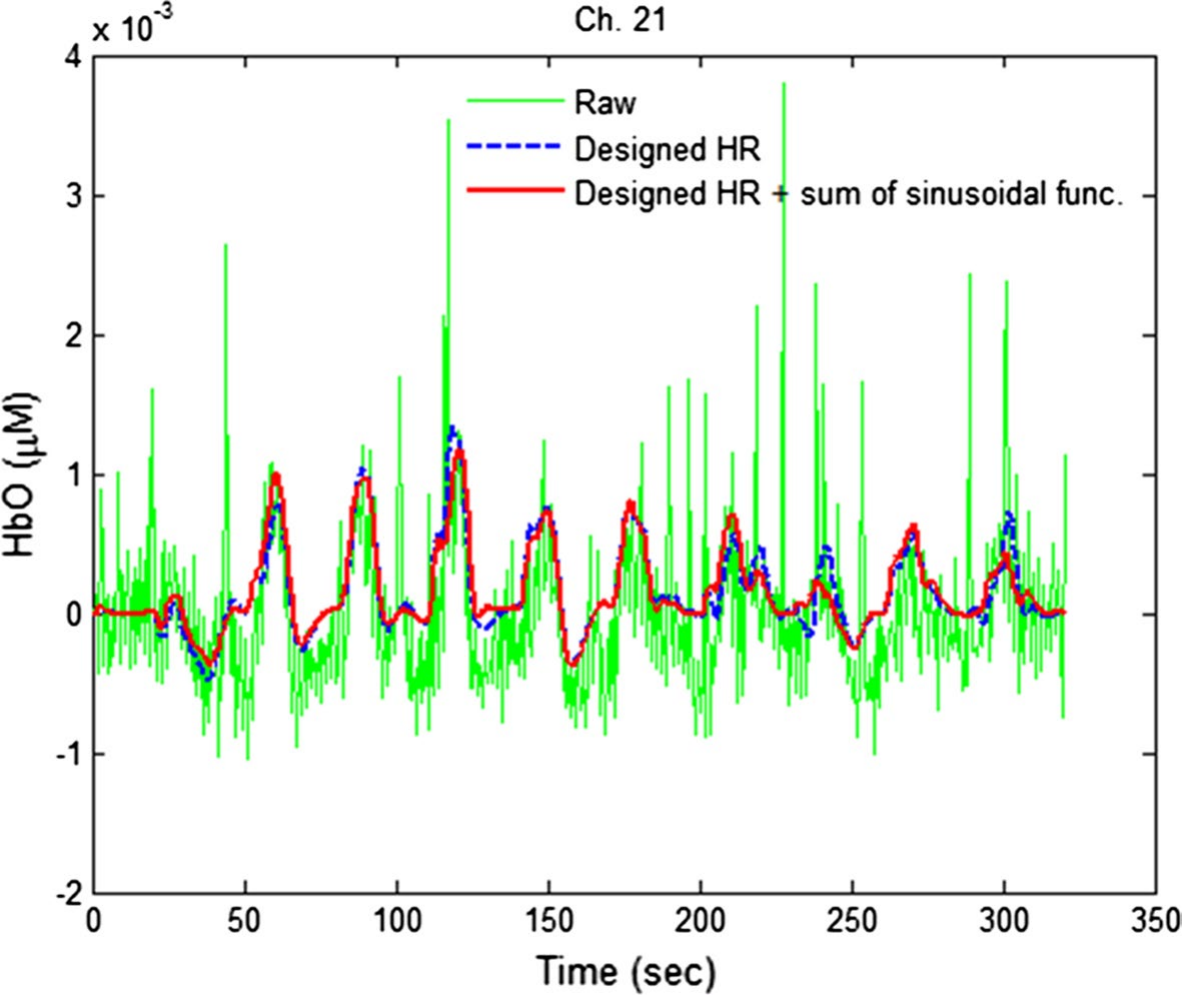
Comparison of HbOs obtained by the proposed model with (red thick curve) and without (blue dashed curve) by using the sum of sinusoidal functions (Ch. 21, Sub.3)

Figure 12 compares the estimated HbOs obtained by the proposed model with and without the addition of the sum of three sinusoidal functions (Ch. 21, Sub. 3) as follows: Ch. 21 was composed of optodes 18 and 30 and was selected because it presented distinguishable results for two methods. The results show that when the physiological noise frequencies are estimated during the 20 s initial resting period prior to the first trial, the proposed method with sinusoidal functions reduced the trial-to-trial variation [65] while the approach without using these terms did not reflect this.

### Comparison with Kalman filter

**Fig. 13:**
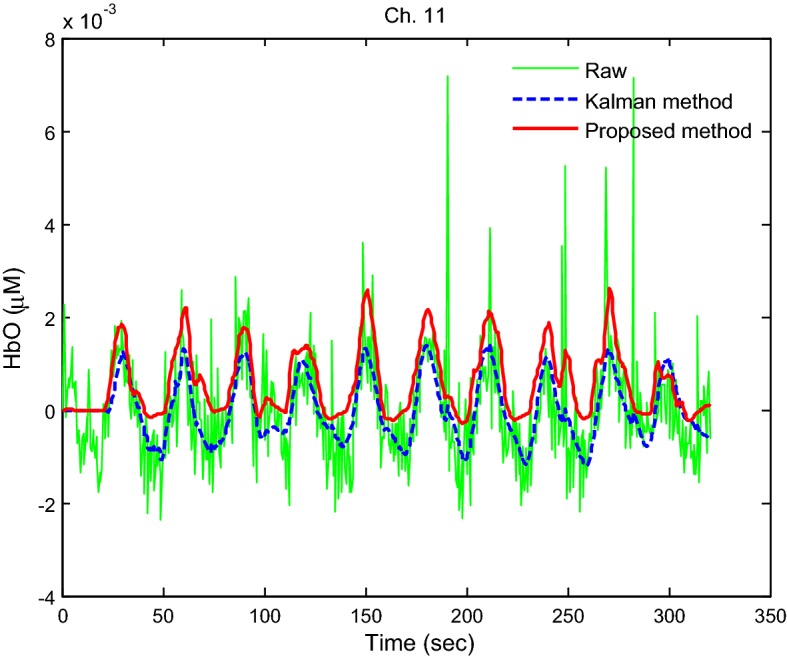
Comparison of the HbOs obtained by the proposed method (red thick curve) and a Kalman filter (blue dashed curve)

Kalman filter is a recursive tracking estimator. The approach estimates the states of a process by using an updated regularized linear inversion scheme [38, 52, 66, 67]. Therefore, the performance of our proposed method was compared with that of a Kalman filter. In the study, the same linear model in Gagnon et al. [40] consisting of two components (canonical HR and SS measurement) is used. It is noted that the canonical HR is a set of 15 Gaussian functions. Additionally, the initial state and process noise covariance matrices were set to identity matrices with diagonal entries of 1 × 10^−1^ and 5 × 10^−4^, respectively, which are the same as those in Gagnon et al. [40]. Figure 13 shows a comparison of the HbOs obtained by a Kalman estimator (blue dashed curve) and the proposed method (red thick curve). The results indicate that our proposed method was comparable with the Kalman filter.

**Fig. 14:**
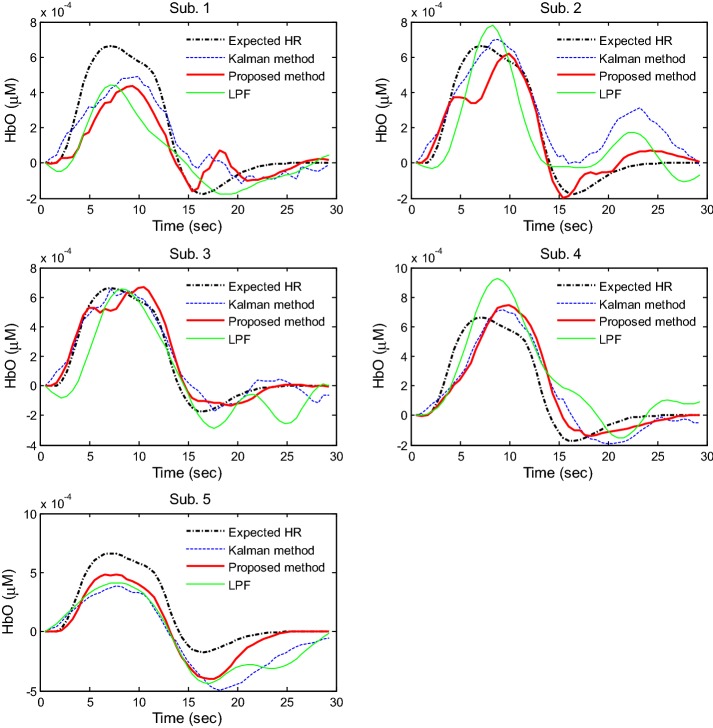
Comparison of the HbOs: Kalman filter, LPF, and the proposed method

To further evaluate the proposed method, the HbOs obtained by three different methods (Kalman filter, LPF, and the proposed method) for five subjects were compared. Figure 14 plots the averaged HbOs over all active channels by using the Kalman filter (blue dashed curves), LPF (green solid curves), and the proposed method (red thick curves). The results suggest that the proposed method is comparable with both the expected HbO (black dotted-dashed curve) and the Kalman-filter-based HbO. A one-way test of variance was performed to evaluate the obtained HRs in Fig. 14. In the case of Kalman filter, the obtained HRs from Sub. 1 indicated significantly different means (*p* = 1.04 × 10^−7^). In the case of LPF, the means of the obtained HRs from Subs. 1 and 4 were significantly different (*p* = 3.1 × 10^−6^). However, the results from the proposed method for all five subjects were not significantly different (*p* = 0.03). This demonstrates that the proposed approach gives the extracted HR more consistently than LPF and Kalman filter methods.

**Fig. 15:**
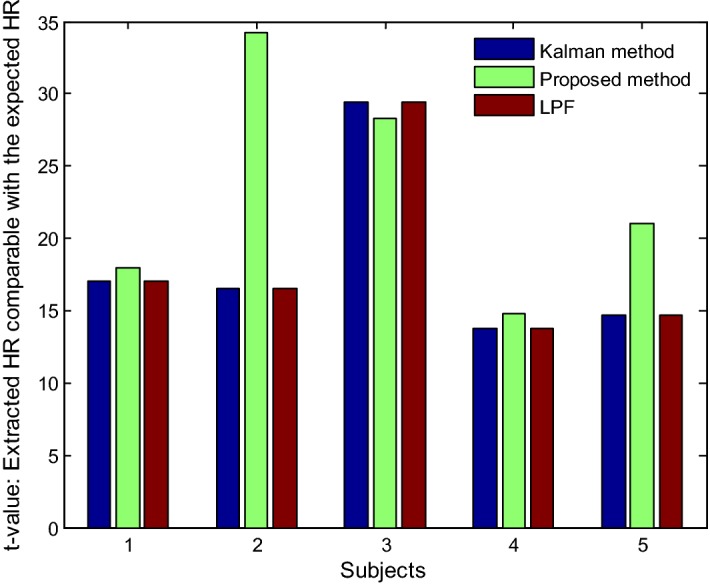
Comparison of *t*-values of the HbOs obtained by three methods (Kalman filter, proposed method, and LPF)

To further compare our proposed method with Kalman filter and LPF methods, *t*-values of the HbOs against the expected HbO were computed by using the *robustfit* function available in MATLAB [58, 59, 68]. As shown in Fig. 15, the *t*-values obtained by using the proposed method exceed those obtained with the Kalman filter and LPF approaches for four out of five subjects.

**Fig. 16:**
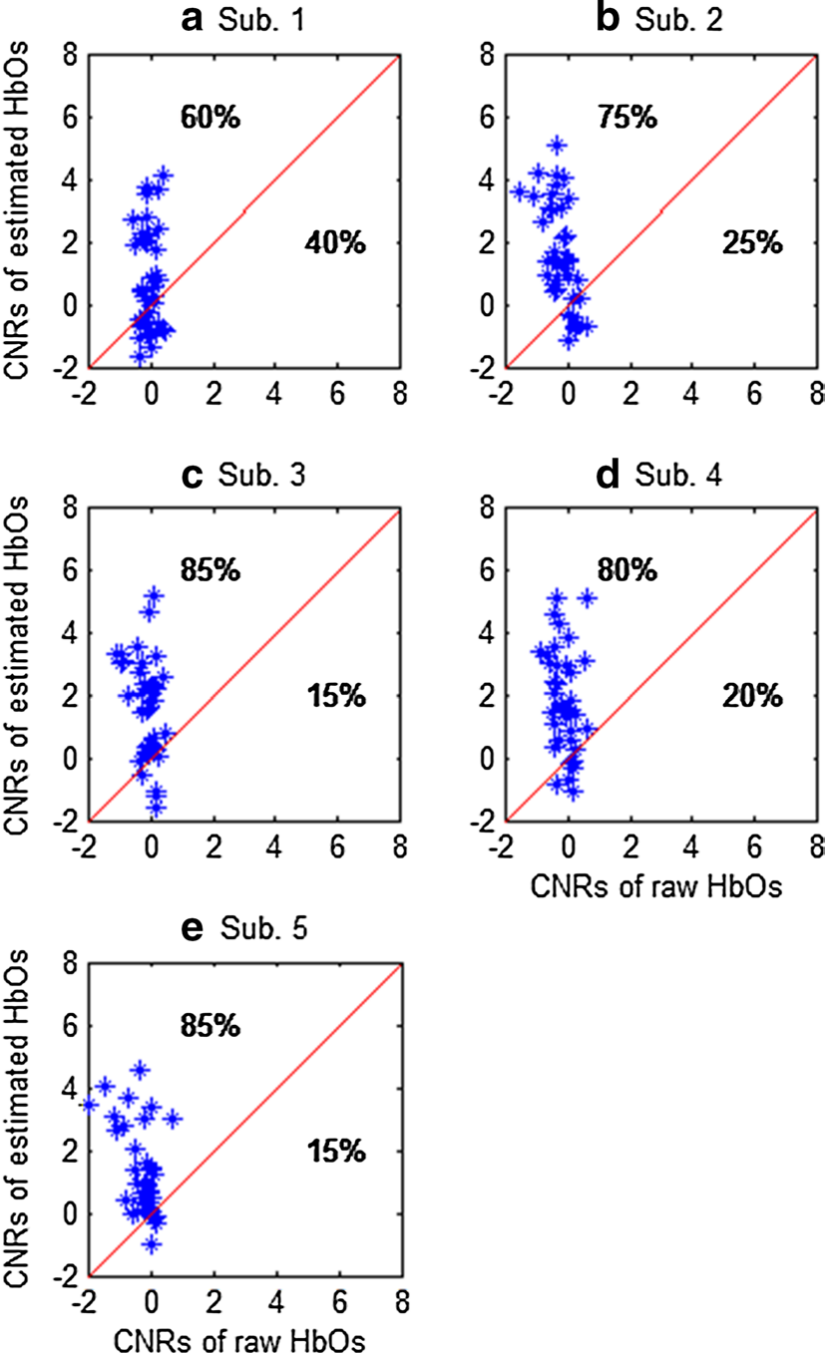
Comparison of CNRs between raw HbOs and the HbOs estimated by the proposed method (the average reduction from five subjects is 77%)

**Fig. 17:**
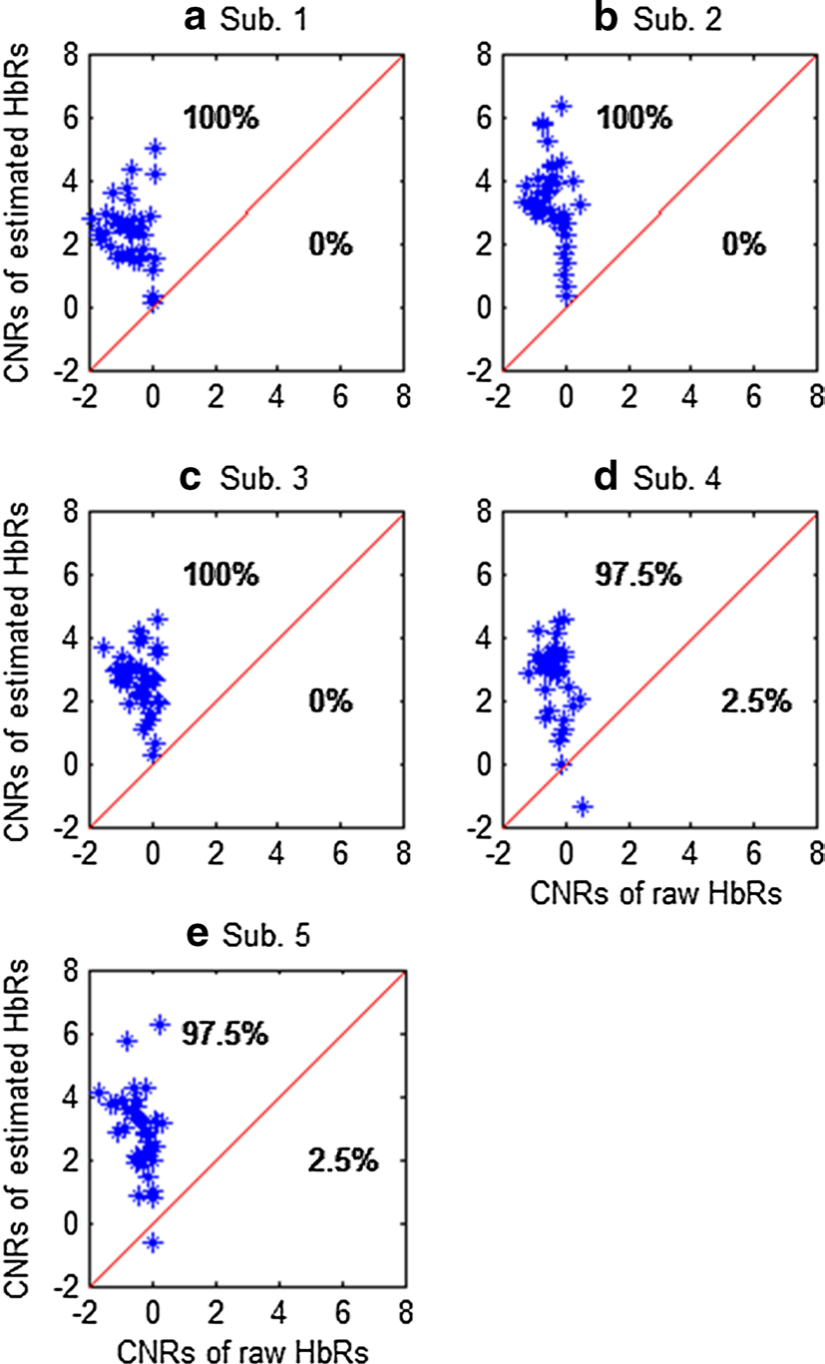
Comparison of CNRs of the raw and filtered HbRs by using the proposed method (average reduction of noise of five subjects: 99%)

Figure 16 compares the CNRs of the raw HbOs and the HbOs obtained by the proposed method across five subjects. The noise-reduction percentages (in terms of the number of channels) of the estimated HbO and HbR in comparison with the raw HbO and HbR were computed by comparing them with the raw fNIRS CNR data. The results indicated that the proposed method demonstrated an average noise reduction of 77% for HbO (see Fig. 16) and 99% for HbR (see Fig. 17). This revealed that the proposed method effectively removed physiological and superficial noises. Additionally, the accuracy of the obtained HRs was significantly improved.

To implement the proposed method for brain imaging, right-finger-movement tasks in the left motor cortex were performed by using a bundled-optode arrangement. In our experiment, the available sampling rate for the bundled arrangement (a total of 32 optodes) was limited to 1.81 Hz. Therefore, with respect to the acquired fNIRS data, the proposed method deduced the cardiac frequency as within approximately 0.8–0.9 Hz. the issues of measuring different brain regions and motion-artifact removal are limited in our current work. Several relevant reports proposed that motion artifacts are measured by means of an accelerometer [2, 22]. If motion artifacts are measured in this manner, then they are included in our model as a new additional component and estimated by the RLSE approach. Specifically, we expect that motion artifacts are effectively reduced in this manner. In addition, in the future works, measured fNIRS data of different brain regions will be checked using our proposed method.

Actually, the proposed method reduces noises online. Therefore, it is appropriate for BCI applications [69–79] based on effective classifiers (e.g., linear discriminant analysis, principle component analysis, and support vector machine) [80–87] and studies on cognitive functions in daily life [88, 89]. The precisely extracted commands from measured data controls external devices if noises are perfectly removed, (i.e., robot arms, wheelchairs, and prosthetic arms) [90, 91].

## Conclusions

In the study, we presented a novel adaptive-filtering-based approach to reduce physiological and superficial noises and decomposition of the HRs in fNIRS data. Our experimental results revealed that the proposed method improved the accuracy of the estimated HR and significantly reduced physiological noises. The averaged noise reductions for HbO and HbR were 77 and 99%, respectively. The results strongly suggest that the proposed model can be utilized for noise removal and HR extraction in both offline and online applications.
